# Altered Hypoxia-Induced and Heat Shock Protein Immunostaining in Secondary Hair Follicles Associated with Changes in Altitude and Temperature in Tibetan Cashmere Goats

**DOI:** 10.3390/ani11102798

**Published:** 2021-09-25

**Authors:** Yanyu He, Xiu Liu, Jie De, Saihong Kang, John S. Munday

**Affiliations:** 1 College of Animal Science and Technology, Agricultural University, Lanzhou 730070, China; y.he@massey.ac.nz; 2 School of Fundamental Sciences, Massey University, Palmerston North 4410, New Zealand; 3 Gansu Key Laboratory of Herbivorous Animal Biotechnology, College of Animal Science and Technology, Gansu Agricultural University, Lanzhou 730070, China; 4 Institute of Animal Sciences, Tibet Academy of Agricultural and Animal Husbandry Sciences, Lhasa 850009, China; deji13659549145@163.com; 5 College of Veterinary Medicine, Gansu Agricultural University, Lanzhou 730070, China; heyanyuxy@sina.com; 6 School of Veterinary Science, Massey University, Palmerston North 4410, New Zealand

**Keywords:** steppe, Tibetan cashmere goat, secondary hair follicle

## Abstract

**Simple Summary:**

Cashmere goats in Tibet are adapted to a high altitude, cold climate, high solar radiation, and hypoxia. The aim of the present study was to compare the morphology of the secondary hair follicles and immunostaining of hair follicle regulatory proteins in Tibetan cashmere goats from a high altitude and low temperature (Rikaze) to goats from a lower altitude and comparatively warm temperature (Huan). We conclude that, at the same time of the year, the secondary hair follicles were at different development stages. HIF-1a protein immunostaining in the inner root sheath (IRS) and hair shaft (HS) was higher than the immunostaining in the outer root sheath (ORS). In contrast, immunostaining for HIF-2a protein in the ORS and IRS was higher than that present in the HS. Immunostaining for HIF-3a protein was higher in the ORS than the IRS while HOXC13 protein immunostaining was higher in the ORS than the IRS and HS. Immunostaining in secondary hair follicles for HIF-1a, HIF-2a, and HSP27 protein in the cashmere goats living in Rikaze was significantly higher than that in the secondary hair follicles of cashmere goats from Huan. In contrast, HOX13 protein immunostaining was significantly higher in cashmere goats from Huan than from Rikaze. These results are useful in understanding how altitude and temperature influence secondary hair follicle development.

**Abstract:**

This experiment compared secondary hair follicles (SFs) in Tibetan cashmere goats from two different steppes that were at different altitudes and had different temperatures. Twenty-four 2-year-old goats were studied. Twelve goats were from Rikaze in Tibet which is at an altitude of above 5000 m with an average temperature of 0 °C. The other 12 studied goats were from Huan County of Gansu Province which is around 2000 m above sea level with an average temperature of 9.2 °C. The structural features of SFs were assessed using light microscopy and transmission electron microscopy. The presence of HIF-1a, HIF-2a, HIF-3a, HSP27, and HOXC13 proteins was studied using immunohistochemistry and immunofluorescence. Light and electron microscopy revealed that the SFs of the Tibetan cashmere goats that lived in the Rikaze Steppe were in the proanagen stage in May. However, the SFs of the goats from the lower warmer Huan County were in the anagen stage at the same time. Immunohistochemistry revealed intense immunostaining for HIF-1a protein in the inner root sheath (IRS) and hair shaft (HS); immunostaining against HIF-2a in the outer root sheath (ORS) and IRS; HIF-3a protein immunostaining in the ORS; HSP27 immunostaining in the ORS, IRS, and HS; and HOXC13 immunostaining in the ORS and HS. HIF-1a protein expression in the IRS and HS was higher than the expression in the ORS (*p* < 0.05) while the expression of HIF-2a protein was higher in the ORS and IRS than the HS (*p* < 0.05). The expression of HIF-3a protein was higher in the ORS than in the IRS (*p* < 0.05). Expression of HOXC13 protein was higher in the ORS than in the IRS and HS (*p* < 0.05). Immunostaining of HIF-1a, HIF-2a, and HSP27 protein was significantly higher in SFs from cashmere goats from Rikaze than in goats from Huan (*p* < 0.05). In contrast, HOX13 protein immunostaining was significantly higher in cashmere goats from Huan than from Rikaze (*p* < 0.05). Significant differences were observed in the SFs of cashmere goats from two locations that differ in altitude and temperature. This suggests the differences in the secondary hair follicles could be due to the hypoxia and lower temperatures experienced by the goats in Rikaze. These results are useful in understanding how altitude and temperature influence SF development. Hair produced by the SFs are used for down fiber. Therefore, understanding of the factors that influence SF development will allow the production and harvest of these valuable fibers to be maximized.

## 1. Introduction

China produces around a half of all cashmere fiber in the world [1]. This fiber is the hair shafts from the secondary hair follicles (SFs) from cashmere goats. Cashmere goats are farmed across mainland China and live in a diverse range of conditions that range from warm sea-level pasture to cold high-altitude (over 5000 m above sea level) farms on the Tibetan Plateau [2].

Due to the value of the cashmere fibers, cashmere goats are economically important animals for farmers on the Tibetan Plateau. Cashmere goats in Tibet have adapted to high altitudes, extreme cold, and hypoxia [3,4,5,6,7,8,9,10,11]. However, goats from the location are famous for producing high-quality down fiber, suggesting the high altitude and cold contribute to the high-quality fiber from these animals.

Hypoxia-inducible factors (HIFs), including HIF-1a, HIF-2a, and HIF-3a, are important in protecting the body against the low-oxygen environment that is present at high altitude [12,13,14]. The expression of HIFs in animals living at high altitude has been reported in many tissues [7,15,16,17]. However, the expression of HIFs in secondary hair follicles has not previously been investigated.

Heat shock protein 27 (HSP27) regulates actin polymerization [18] and has been found to be expressed more in anagen hair follicles than in telogen hair follicles [19] and expression of this protein is also correlated to epidermal differentiation in human skin [20]. In addition, in Longdong cashmere goats, HSP27 immunostaining in secondary hair follicles was found to be different in extensively fed animals compared to those that were in an intensively fed group [21].

HOXC13 is also involved in hair follicle formation and growth [22,23] and this protein has been shown to be expressed in the epidermis and outer root sheath (ORS) of SFs and correlated with cashmere goat skin thickness [24,25].

The purpose of the presently reported experiment is to compare the SFs of cashmere goats from a high altitude and cold climate to the follicles of cashmere goats from a lower altitude and more moderate temperatures. To do this, goats from the Rikaze Steppe (an average altitude of over 4000 m with a yearly average temperature of 0 °C) were compared to goats from the Huan County Steppe (an average altitude of 2000 m and a yearly average temperature of 9.2 °C). The presence of HIF-1a, HIF-2a, HIF-3a, HSP27, and HOXC13 in the SFs in samples taken from Tibetan cashmere goats from Rikaze was compared to the presence of these proteins in SFs from goats from Huan. The presence of differences would enable greater understanding of how goats respond to hypoxia and changes in temperature and may inform methods to improve Tibetan cashmere goat down fiber production. To the authors’ knowledge, differences in the SFs of cashmere goats from different environments have not been previously studied.

## 2. Materials and Methods

### 2.1. Tibet Cashmere Goats in Rikaze Steppe and Huan County Steppe

Twenty-four two-year-old non-pregnant female cashmere goats were studied. Twelve goats were from the Rikaze Steppe (29°15′0″ N, 88°52′59″ E) of Tibet while the others were from Huan County (36°35′59.99″ N, 107°05′60.00″ E) in Gansu. The goats from Huan County had been born in the same herd as the goats from Rikaze, but had been transported to Huan when they were 1 year old. All goats were grazed on pasture.

### 2.2. Skin Sample Collection

A sample of skin was removed from the same area from all goats. The area sampled was from the dorsum at the level of L2. All samples were taken over 3 days in May 2020 immediately after slaughter at a commercial abattoir that conformed with all regional regulations regarding the slaughter process. Collected samples were preserved in formalin for light microscopy and modified Karnovsky’s fixative (3% gluteraldehyde (*v*/*v*) 2% formaldehyde (*w*/*v*) in 0.1 M phosphate buffer (pH 7.2)) for samples that were to be used for electron microscopy.

### 2.3. Wax Section and Ultrathin Section Analysis

Hematoxylin and eosin sections of the skin samples were prepared following standard methods. Samples were prepared and examined by transmission electron microscopy (TEM) as previously described [25].

### 2.4. Immunohistochemical and Immunofluorescence Analysis

For immunohistochemistry, 5 µm tissue sections were mounted on charged slides and antigen retrieval was performed in 10 mM citrate buffer (pH 6.0) for 30 min in a microwave oven. After washing in PBS, the sections were preincubated for 60 min at room temperature in 5% goat serum, and incubated overnight at 48 °C with primary antibody against rabbit HIF-1a, HIF-2a, HIF-3a, and HOXC13 (all antibodies diluted 1:200, Bioss, China, bs-20398R, bs-1447R, bs-5989R, and bs-13599R) and primary antibody against mouse HSP27 (ab-2790 Abcam, Hong Kong) overnight at 4 °C. After washing five times for 5 min with PBS, the sections were incubated in rabbit and mouse Histostain-Plus Kits (SP-0022 and SP-0024, Bioss, Beijing, China), respectively, and antibodies were visualized using diaminobenzidine (DAB, Bioss, Beijing, China) with a Meyer’s hematoxylin counterstain. To detect immunofluorescence, 5 µm tissues sections were rehydrated and incubated in PBS with antibodies against rabbit HIF-1a, HIF-2a, HIF-3a, HOXC13, or mouse HSP27 (as described for immunohistochemistry except diluted 1:100 overnight at 4 °C), then the fluorescent secondary antibodies (donkey anti-mouse, Abcam, Hong Kong, GR112688-1/donkey anti-rabbit, Abcam, Hong Kong, GR115771-1) were added at a dilution of 1:1000 in PBS. The primary antibody was replaced with PBS for negative controls. The stained slides were examined and photographed using CaseViewer.

### 2.5. Measurements and Statistical Analysis

To quantify the integrated optical density (IOD) of immunoreactivity of sections of secondary follicles, images of immunostaining sections were taken and the IOD measured using Image Pro-Plus 6.0 software (Media Cybernetics, Inc., Bethesda, MD, USA) as previously described [15]. The data were expressed as the mean ± SD and were analyzed by one-way ANOVA using SPSS software (version 17.0). A *p*-value of <0.05 was considered statistically significant. An independent sample *t*-test using was used to compare the optical densities of immunostaining from goats from Rikaze Steppe with goats from Huan County. A *p*-value of <0.05 was considered statistically significant.

## 3. Results

### 3.1. Morphological Analysis of Secondary Hair Follicle and Histomorphological Evaluations

**Figure 1:**
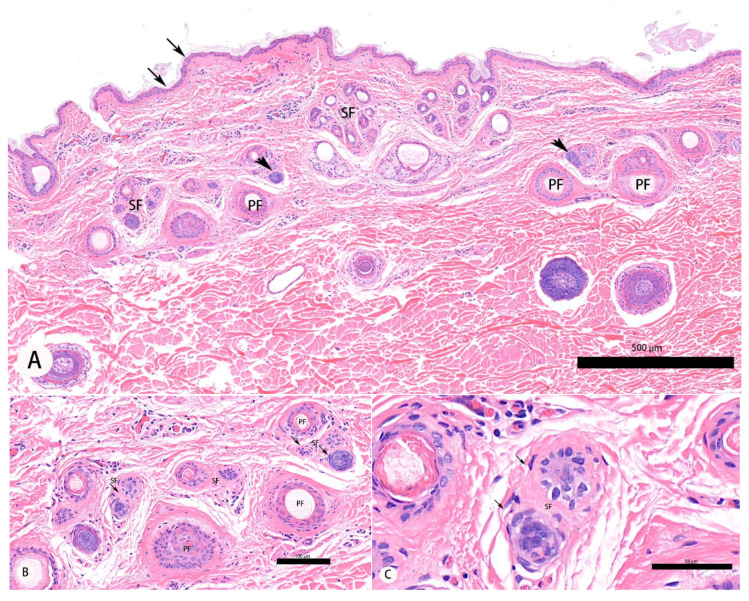

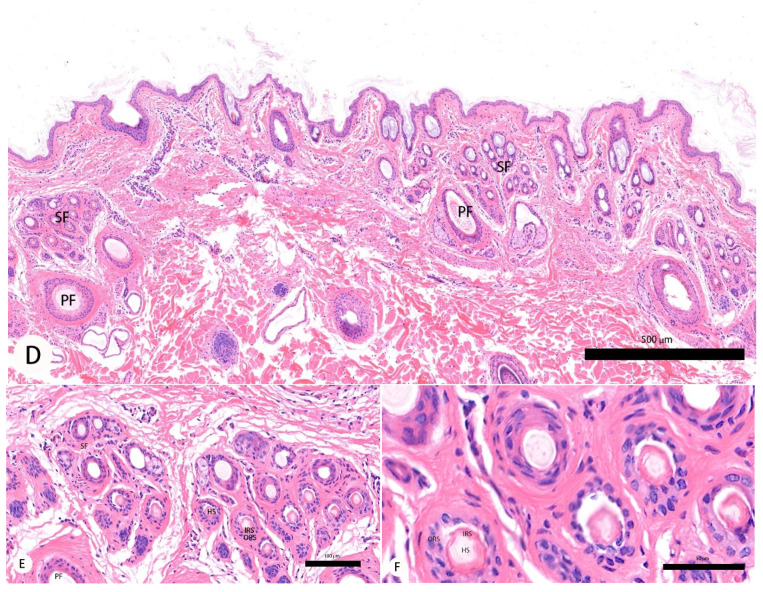
Photomicrographs of secondary hair follicles of cashmere goats. (**A**) Section of skin from a goat from Rikaze. Arrows indicate the epidermis while arrowheads indicate finger cell groups. (**B**,**C**) Secondary hair follicles of a goat from Rikaze. The secondary hair follicles are in the proanagen stage. (**D**) Section of skin from a goat from Huan. The arrows indicate the epidermis. (**E**,**F**) Secondary hair follicles of a goat from Huan County. The secondary hair follicles are in the anagen phase. PF: primary hair follicle; SF: secondary hair follicle; HS: hair shaft; IRS: inner root sheath; ORS: outer root sheath. (**A**,**D**) 100×; (**B**,**E**) 200×; (**C**,**F**) 400×.

At the time that the samples were taken (May), SFs from cashmere goats in Rikaze were in the proanagen phase while these hair follicles from goats in Huan County were in the anagen phase of growth (Figure 1A–C). Using light microscopy, hair follicles in the anagen phase were characterized by a high density of SFs (Figure 1D,E), a rounded morphology of the hair bulbs (Figure 1F), and the presence of inner root sheaths (IRSs) and ORSs. Hair bulbs showed complete division; the cells of the dermal papilla had high-density pear-shaped granules (Figure 1F).

**Figure 2:**
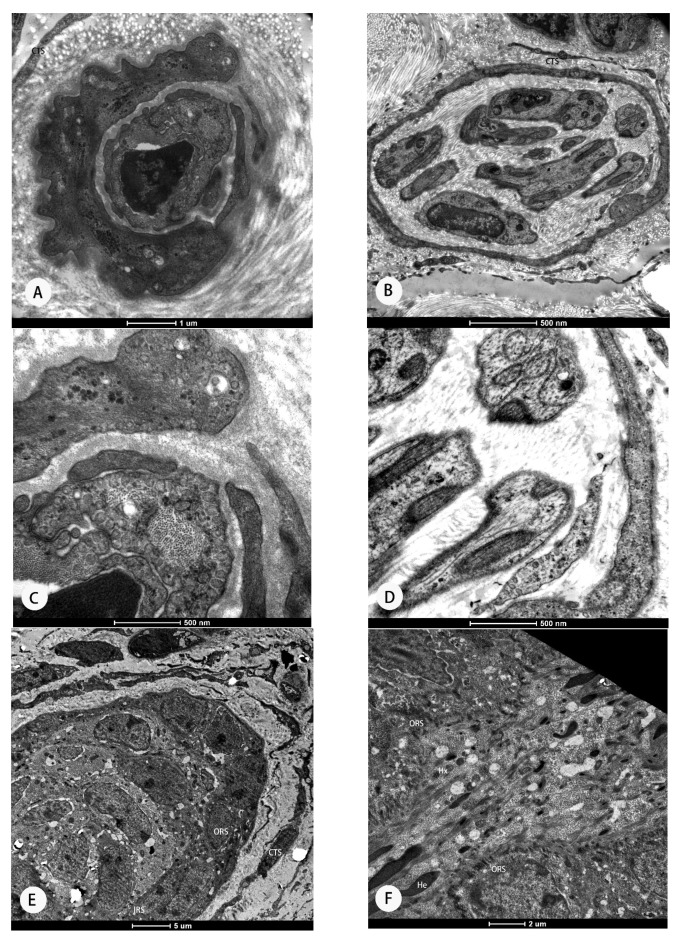

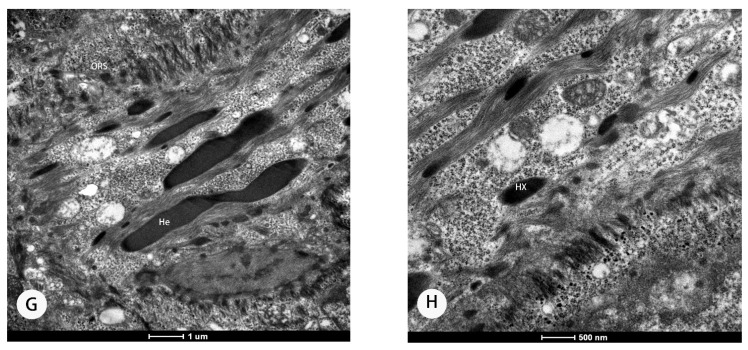
The secondary hair follicles of cashmere goats from Rikaze and Huan County under an electron microscope. (**A**–**D**) Ultrastructure of new fiber tip of proanagen secondary hair follicle from a goat from Rikaze. (**E**–**H**) Ultrastructure of anagen phase secondary hair follicle from a goat from Rikaze. ORS: outer root sheath; IRS: inner root sheath; CTS: connective tissue sheath; HX: Huxley’s layer; He: Henle’s layer.

Using TEM, the proanagen phase was identified by the hair follicle rudiments which were visible and surrounded by the cells of the ORS (Figure 2A,B). The elongation zone contained undifferentiated keratinocytes (Figure 2C,D). TEM revealed that the IRS consisted of three concentric layers which is the typical ultrastructure of anagen secondary hair follicle (Figure 2E–H), including Huxley’s layer and Henle’s layer, which was the outermost layer (Figure 2F,G).

### 3.2. Immunohistochemical Detection of HIF-1a, HIF-2a, HIF-3a, HSP27, and HOXC13 in Secondary Hair Follicles

**Figure 3:**
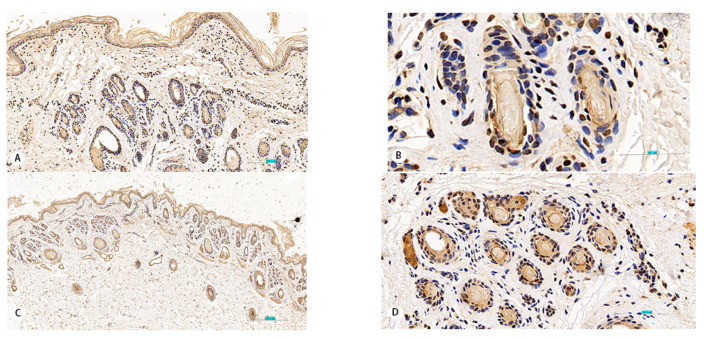

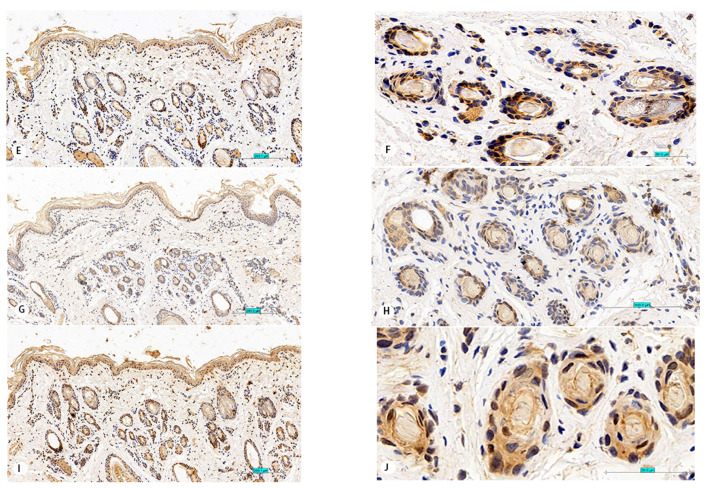
Immunostaining of HIF-1a, HIF-2a, HIF-3a, HSP27, and HOCX13 in secondary hair follicles of Tibetan cashmere goats. (**A**,**B**) HIF-1a immunostaining is present in the inner root sheath and hair shaft. (**C**,**D**) HIF-2a immunostaining in outer root sheath and inner root sheath. (**E**,**F**) HIF-3a immunostaining in outer root sheath. (**G**,**H**) HSP27 immunostaining in outer root sheath, inner root sheath, and hair shaft. (**I**,**J**) HOCX13 immunostaining in outer root sheath and hair shaft. (**A**,**C**,**E**,**G**,**I**) 100×; (**D**,**F**,**H**) 200×; (**B**,**J**) 400×.

Immunostaining for HIF-1a, HIF-2a, HIF-3a, HSP27, and HOXC13 was present in the ORS, IRS, and HS in samples from both groups of cashmere goats. Intense immunostaining for HIF-1a protein was present in the IRS and HS (Figure 3A,B), HIF-2a immunostaining was visible in the ORS and IRS (Figure 3C,D), while HIF-3a immunostaining was visible in the ORS (Figure 3E,F). HSP27 expression was present in the ORS, IRS, and HS (Figure 3G,H). However, HOXC13 expression was only visible in the ORS and HS (Figure 3I,J).

**Figure 4:**
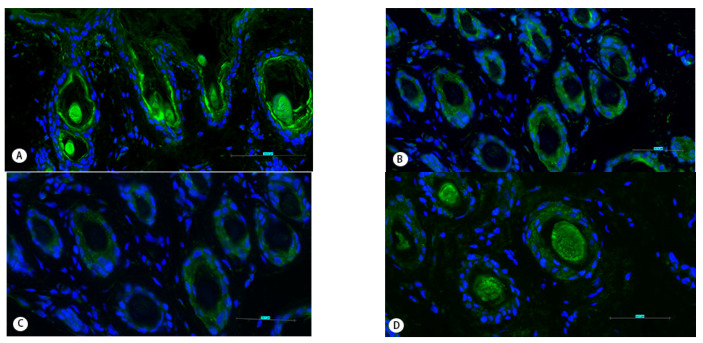

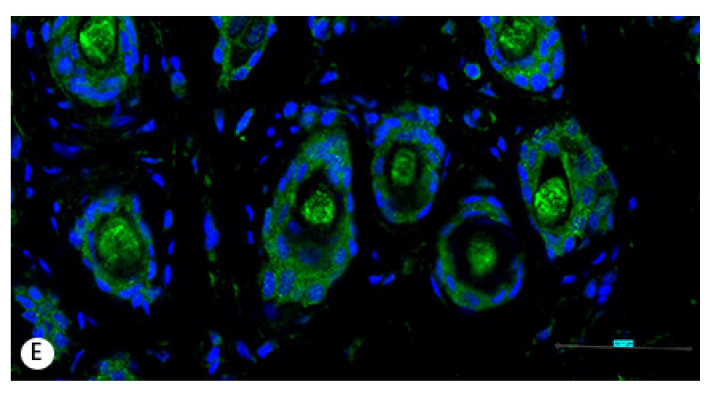
Immunofluorescence detection of HIF-1a, HIF-2a, HIF-3a, HSP27, and HOCX13 in secondary hair follicles of Tibetan cashmere goats. (**A**) HIF-1a is visible in inner root sheath and hair shaft. (**B**) HIF-2a immunostaining in outer root sheath and inner root sheath. (**C**) HIF-3a immunostaining in outer root sheath. (**D**) HSP27 is visible in outer root sheath, inner root sheath, and hair shaft. (**E**) HOCX13 is visible in outer root sheath and hair shaft. (**A**–**E**) 400×.

HIF-1a immunofluorescence was present in the IRS and HS (Figure 4A) while HIF-2a was present in the ORS and IRS (Figure 4B). HIF-3a was found in the ORS (Figure 4C), and HSP27 was found in the ORS, IRS, and HS (Figure 4D). The immunofluorescence of HOXC13 was found in the ORS and HS (Figure 4E).

**Figure 5:**
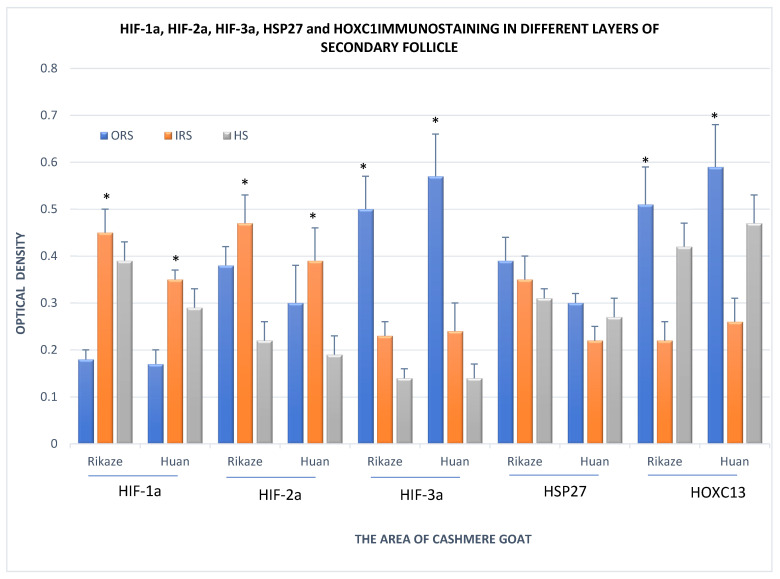
Immunostaining of HIF-1a, HIF-2a, HIF-3a, HSP27, and HOXC13 in outer root sheath, inner root sheath, and hair shaft of the secondary hair follicle in the Tibetan cashmere goat. Values are means ± SD (*n* = 12). Red bars indicate the outer root sheath, orange bars the inner root sheath, and yellow bars the hair shaft. Rikaze indicates the goats lived in the Rikaze Province while Huan indicates the goats were from Huan County. * Indicates the presence of significant differences (*p* < 0.05) in the IOD values between goats from Rikaze and goats from Huan in that protein in that layer of the secondary hair follicle.

Optical density analysis revealed that more intense HIF-1a immunostaining was present in the IRS and HS than in the ORS (*p* < 0.05), there was higher HIF-2a protein immunostaining in the ORS and IRS than the in HS (*p* < 0.05), higher HIF-3a protein immunostaining in the ORS than the IRS (*p* < 0.05), and HOXC13 immunostaining was higher in the ORS than the in IRS and HS (*p* < 0.05; Figure 5). However, there were no significant differences in the immunostaining of HSP27 protein between the ORS, IRS, and HS.

**Figure 6:**
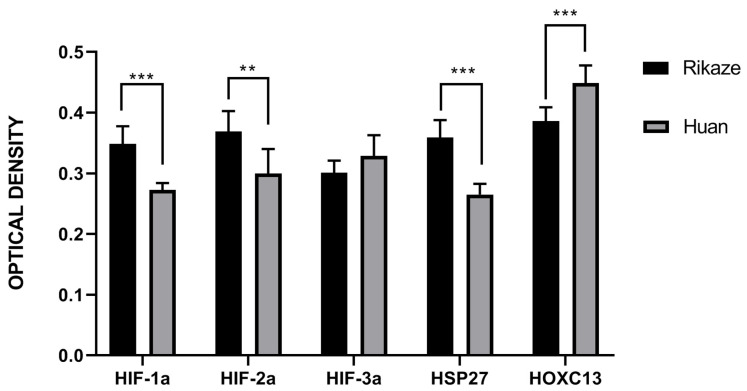
Total immunostaining of HIF-1a, HIF-2a, HIF-3a, HSP27, and Hoxc13 in the secondary hair follicles of Tibetan cashmere goat. Rikaze indicates that the goat was from Rikaze Province while Huan indicates the goat was from Huan County. ** Indicates a significant difference in which the *p*-value was between 0.01 and 0.05 while *** indicates a significant difference with a *p*-value < 0.01.

When the total immunostaining throughout the SFs in goats from Rikaze was compared to the total immunostaining in the SFs from goats from Huan, total immunostaining for HIF-1a, HIF-2a, and HSP27 protein was higher in hair follicles from Rikaze goats than Huan goats (*p* < 0.05; Figure 6). However, there was less HOX13 protein immunostaining in the hair follicles of goats living in Rikaze compared to those of goats from Huan County (*p* < 0.05).

## 4. Discussion

In the presently described experiment, all twenty-four Tibetan cashmere goats were born in Rikaze and lived at high altitude in a cold climate for their first year of life. However, twelve of these goats were then subsequently moved to a lower altitude and a warmer climate for a year prior to samples of the skin being taken. This was to observe what differences in the SFs were present between the two groups of goats. This would allow evaluation of some of the adaptations made by the goats in response to the lower altitude and warmer climate. Evaluation of the SFs revealed that there were significant differences between Tibetan cashmere goats from different steppes. The SFs in goats from Huan County were in anagen, suggesting the hair follicles developed faster than the hair follicles of goats from the higher, colder Rikaze Province. This suggests hypoxia and low environmental temperature could delay entry of the SF into the anagen stage. These results support previous studies that suggested growth of SFs is influenced by the sunshine period and environmental temperature [21,26]. They also suggest that a warm environment and lower altitude may promote the growth of cashmere down fiber.

The heat shock proteins influence cell growth and differentiation and HSP expression increases in response to heat, oxidative stress, or glucose deprivation [27]. HSP27 has previously been shown to be expressed in the epidermis [28,29,30] with levels of expression of this protein increasing with increased epidermal differentiation and trichilemmal keratinization [20]. A study of cashmere goats revealed that HSP27 protein expression may be influenced by the level of nutrition and, therefore, this protein may allow adaptation to adverse environment conditions [21]. In the present study, it was shown that HSP27 was present in all layers of the SFs, and the expression of HSP27 in Rikaze was higher than them in Huan County. The harsh environment of Rikaze compared with Huan appeared to increase expression of this protein. This suggests that HSP27 expression in SFs may be influenced by environment stress. HOXC13 expression has been shown to be associated with goat hair follicle formation and hair growth [21,22], with protein expression correlated with cashmere goat skin thickness [24]. In addition, the expression of HOXC13 appeared to be correlated to the activity of the SF, suggesting a role of this protein in stimulating new hair follicle development [21]. In the present research, it was found that HOCX13 was mainly present in the ORS, and the expression was higher in goats from lower, hotter places than in goats from a harsher environment. These results indicate that HOXC13 expression is not induced by cold or hypoxia, although this protein also appears important in regulating development of the SFs.

In the present experiment, HIF-1a, HIF-2a, and HIF-3a immunostaining was present in all layers of the SFs. However, the immunostaining of HIF-1a was highest in the IRS and HS, HIF-2a was highest in the ORS and IRS, and HIF-3a was highest in the ORS. It is speculated that HIF-1a has a key role in hair shaft growth, and HIF-2a and HIF-3a influence secondary hair follicle reconstruction. Comparing the HIF immunostaining between the two groups of cashmere goats showed that HIF-1a and HIF-2a were higher in goats living in the harsher Rikaze Steppe compared to goats from Huan County. This suggests that HIF-1a and HIF-2a may be involved in the response to the harsh environment stress of SF development. The immunostaining of HIF-1a and HIF-2a proteins in SFs between two groups has the same trend, which suggests the two isoforms may be regulated by similar factors. However, the immunostaining of HIF-3a in the goats that lived in Rikaze was lower than in goats from Huan County. This may suggest that, although HIF-3a influences the development of the SFs, expression of this protein is not increased by a harsh environment. These results are consistent with a previous study that also found differences in HIF-3a expression due to altitude-induced hypoxia [16]. The Tibetan cashmere goats in Huan County were in anagen stage, which is the phase at which the highest development of SFs occurs. As the immunostaining of HIF-3a and HOCX13 was higher in goats from Huan County, it is possible that HIF-3a and HOXC13 may influence the development of SFs within the anagen phase.

## 5. Conclusions

The results of this study suggest: (1) Hypoxia and environmental temperature may influence SF development and may influence entry of the follicle into the anagen phase. (2) HSP27, HIF-1a, HIF-2a, and HIF-3a have a role in the response of the hair follicle to adverse environmental conditions. (3) HOXC13 expression was not increased in animals subject to a harsh environment.
